# Supplementary material for: Analysis of competitive and comparative advantages of potato production in Indonesia
Source: PLoS One. 2022 Feb 24;17(2):e0263633. doi: 10.1371/journal.pone.0263633 (PMC8870507; doi:10.1371/journal.pone.0263633)
Supplement: S1 File — (DOCX) [file pone.0263633.s001.docx]

**SUPPORTING INFORMATION:**

| S1. Allocation of costs into tradable inputs and domestic factor components of potato farming in Indonesia’s producing areas, Dry Season 2019 – 2019/2020 | | | | | | | | | | | |
| --- | --- | --- | --- | --- | --- | --- | --- | --- | --- | --- | --- |
| **No.** | | | **Cost types** | | **Tradable Inputs (%)** | | | **Domestic Factor (%)** | | | |
| **A** | | | **Production Input** | |  | | |  | | | |
| 1 | | | Potato seed | | 100 | | | 0 | | | |
| 2 | | | Fertilizer and Dolomite | |  | | |  | | | |
|  | | | a. Urea/ZA | | 100 | | | 0 | | | |
|  | | | b. SP-36/TSP | | 100 | | | 0 | | | |
|  | | | c. KCl/KNO3 | | 100 | | | 0 | | | |
|  | | | d. NPK/PHONSKA | | 100 | | | 0 | | | |
|  | | | e. Solid organic fertilizer | | 0 | | | 100 | | | |
|  | | | f. Liquid organic fertilizer | | 0 | | | 100 | | | |
|  | | | g. Dolomite | | 0 | | | 100 | | | |
| 3 | | | Pesticide | | 100 | | | 0 | | | |
| 4 | | | Mulch | | 100 | | | 0 | | | |
| 5 | | | Crop stand | | 0 | | | 100 | | | |
| **B** | | | **Labour** | |  | | |  | | | |
| 1 | | | Pre harvest | | 0 | | | 100 | | | |
| 2 | | | Harvest & transport | | 22.50 | | | 77.50 | | | |
| 3 | | | Post-harvest | | 25.00 | | | 75.00 | | | |
| **C** | | | **Depreciation of tools** | | 22.50 | | | 77.50 | | | |
| **D** | | | **Land lease** | | 0.00 | | | 100.00 | | | |
| **E** | | | **Other costs** | | 0.00 | | | 100.00 | | | |
| **F** | | | Capital costs | | 0.00 | | | 100.00 | | | |
| S2a. Shadow prices of inputs and outputs of potato farming in Indonesian upland  agroecosystem in Dry Season, 2019 (Rp/unit | | | | | | | | | | | |
| No | Description | | Bandung | | Wonosobo | Pasuruan | | Karo Land | solo | Kerinci | |
| A | Production Input | |  | |  |  | |  |  |  | |
| 1 | Seeds (Rp/kg) | | 22,388 | | 23,850 | 20,700 | | 22,500 | 22,500 | 22,500 | |
| 2 | Organic fertilizer (kg) | |  | |  |  | |  |  |  | |
|  | 1. Solid organic fertilizer | | 1,000 | | 680 | 500 | | 500 | 750 | 680 | |
|  | 1. Liquid organic fertilizer | | 85,000 | | 65,000 | 67,500 | | 70,000 | 87,500 | 70,000 | |
| 2 | Inorganic fertilizer and lime (Rp/Kg) | |  | |  |  | |  |  |  | |
|  | a. Urea/ZA | | 2.145 | | 2,315 | 2,200 | | 2,227 | 2,282 | 2,362 | |
|  | b. SP-36/TSP | | 3,517 | | 3,687 | 3,582 | | 3,622 | 3,677 | 3,757 | |
|  | c. KCl/KNO3 | | 4,605 | | 4,705 | 4,695 | | 4,445 | 4,445 | 4,445 | |
|  | d. NPK | | 5,921 | | 6,091 | 5,986 | | 5,946 | 6,081 | 6,161 | |
|  | e. Dolomite | | 1,000 | | 1250 | 1,000 | | 1,000 | 700 | 1,000 | |
| 3 | Pesticides (Rp/ha) | | 14,450,000 | | 7,160,000 | 15,600,000 | | 6,800,000 | 5,952,000 | 6,800,000 | |
| 4 | Mulch (Rp/ha) | | 600,000 | | 600,000 | 620,000 | | 600,000 | 620,000 | 600,000 | |
| 5 | Stake (Rp/ha) | | 385 | | 186 | 285 | | 400 | 400 | 190 | |
| B | Labour (Rp/day) | |  | |  |  | |  |  |  | |
| 1 | Pre-Harvest | | 70,000 | | 75,000 | 70,000 | | 80,000 | 85,208 | 75,000 | |
| 2 | Harvest | | 70,000 | | 75,000 | 70,000 | | 80,000 | 85,208 | 75,000 | |
| 3 | Post-harvest | | 70,000 | | 75,000 | 70,000 | | 80,000 | 85,208 | 75,000 | |
| C | Irrigation Fee (Rp/ha/season) | | 5,250,000 | | 3,560,000 | 3,560,000 | | 4,265,000 | 4,680,000 | 4,675,000 | |
| D | Equipment Depreciation Cost | | 996,667 | | 1,050,000 | 642,000 | | 1,725,000 | 1,840,000 | 1,640,000 | |
| E | Land Rent (Rp/ha/season) | | 9,750,000 | | 7,500,000 | 6,500,000 | | 6,000,000 | 6,500,000 | 6,000,000 | |
| F | Other Fees | | 450,000 | | 1,050,000 | 560,000 | | 475,000 | 420,000 | 420,000 | |
| G | Capital Interest (Rp/ha/season) | |  | |  |  | |  |  |  | |
| H | Potato output (Rp/kg) | | 9,306 | | 9,476 | 9,371 | | 9.411 | 9,466 | 9.546 | |

| S2b. Shadow prices of inputs and outputs of potato farming in Indonesian upland agroecosystem in in Rain Season, 2019-2020 (Rp/unit) | | | | | | | |
| --- | --- | --- | --- | --- | --- | --- | --- |
| No | Description | Bandung | Wonosobo | Pasuruan | Karo Land | solo | Kerinci |
| A | Production inputs |  |  |  |  |  |  |
| 1 | Seed (Rp/kg) | 22,500 | 22,500 | 20,700 | 22,500 | 22,500 | 22,500 |
| 2 | Organic fertilizer (kg) |  |  |  |  |  |  |
|  | 1. Solid organic fertilizer | 1,000 | 600 | 500 | 500 | 750 | 600 |
|  | 1. Liquid organic fertilizer | 85,000 | 65,000 | 65,000 | 70,000 | 87,500 | 70,000 |
| 2 | Inorganic fertilizer and lime (Rp/Kg) |  |  |  |  |  |  |
|  | a. Urea/ZA | 2.145 | 2,315 | 2,200 | 2,227 | 2,282 | 2,362 |
|  | b. SP-36/TSP | 3,517 | 3,687 | 3,582 | 3,622 | 3,677 | 3,757 |
|  | c. KCL/KNO3 | 4,605 | 4,705 | 4,695 | 4,445 | 4,445 | 4,445 |
|  | d. NPK | 5,921 | 6,091 | 5,986 | 5,946 | 6,081 | 6,161 |
|  | e. Dolomite | 1,000 | 1,250 | 1,000 | 1,000 | 700 | 1,000 |
| 3 | Pesticides (Rp/ha) | 11,560,000 | 14,040,000 | 15,600,000 | 8,640,000 | 6,912,000 | 6,800,000 |
| 4 | Mulch (Rp/ha) | 640,000 | 620,000 | 620,000 | 600,000 | 560,000 | 620,000 |
| 5 | Stake (Rp/ha) | 400 | 190 | 285 | 500 | 500 | 190 |
| B | Labour (Rp/day) |  |  |  |  |  |  |
| 1 | Pre-Harvest | 70,000 | 75,000 | 70,000 | 80,000 | 85,208 | 75,000 |
| 2 | Harvest | 70,000 | 75,000 | 70,000 | 80,000 | 85,208 | 75,000 |
| 3 | Post-harvest | 70,000 | 75,000 | 70,000 | 80,000 | 85,208 | 75,000 |
| C | Irrigation Fee (Rp/ha/season) | 2,625,000 | 1,789,000 | 1,780,000 | 2,132,500 | 2,340,000 | 2,337,500 |
| D | Depreciation | 996,667 | 1,050,000 | 642,000 | 2,132,500 | 1,840,000 | 1,640,000 |
| E | Land Rent (Rp/ha/season) | 9,750,000 | 7,500,000 | 6,500,000 | 6,000,000 | 6,500,000 | 6,000,000 |
| F | Other Fees | 333.333 | 520,000 | 560,000 | 475,000 | 460,000 | 420,000 |
| G | Capital Interest (Rp/ha/season) |  |  |  |  |  |  |
| H | Potato output (Rp/kg) | 9,306 | 9,476 | 9,371 | 9.411 | 9,466 | 9.546 |

| S3a. The results of the Policy Analysis Matrix (PAM) analysis of potato farming in Bandung Regency, West Java Province, Dry Season 2019 | | | | |  |  |
| --- | --- | --- | --- | --- | --- | --- |
| Variables | Revenue | Cost | | Profit |  |  |
|  |  | *tradable input* | *Domestic factor* |  |  |  |
| Private price | 196.822,500 | 91,101,160 | 75,345,698 | 30,375,642 |  |  |
| Social Price | 227,531,700 | 80,285,619 | 73,438,624 | 73,807,457 |  |  |
| Policy and divergence impacts | -30,709,200 | 10,815,541 | 1,907.074 | -43,431,814 |  |  |
| 1. Private Profitability (PP): D = A – (B + C); | | |  | 30,375,642 |  |  |
| 2. Social Profitability (SP): H = E – (F + G); | | |  | 73,807,457 |  |  |
| 3. Private Cost Ratio : PCR = C/(A – B); | | |  | 0.713 |  |  |
| 4. Domestic Resource Cost Ratio : DRCR = G / (E – F); | | | | 0.499 |  |  |
| 5. Output Transfer : OT = A – E; | | |  | -30,709,200 |  |  |
| 6. Nominal Protection Coefficient on Tradable Output : NPCO = A/E; | | | | 0.865 |  |  |
| 7. Input Transfer : IT = B – F; | |  |  | 10,815,541 |  |  |
| 8. Nominal Protection Coefficient on Tradable Input : NPCI = B / F; | | | | 1.135 |  |  |
| 9. Transfer factor : FT = C – G; | | |  | 1,907.074 |  |  |
| 10. Effective Protection Coefficient : EPC = (A – B) / (E – F); | | | | 0.718 |  |  |
| 11. Net Transfer : NT = D – H; | |  |  | -43,431,814 |  |  |
| 12. Profitability Coefficient LPC = D/H; | | |  | 0.412 |  |  |
| 13. Subsidy Ratio to Producer : SRP = L/E. | | |  | -0.191 |  |  |
| S3b. The results of the Policy Analysis Matrix (PAM) for potato farming in Bandung Regency, West Java Province, Rain Season, 2019-2020 | | | | | | |
| Variables | | Revenue | Cost | | Profit | |
|  |  |  | *tradable input* | *Domestic factor cost* |  |  |
| Private price | | 204,670,950 | 91,101,160 | 75,345,698 | 38,224,092 | |
| Social Price | | 233,701,578 | 85,908,887 | 71,434,962 | 76,357,729 | |
| Policy and divergence impacts | | -29,030,628 | 5,192,273 | 3,910,736 | -38,133,637 | |
| 1. Private Profitability (PP): D = A – (B + C); | | | |  | 38,224,092 | |
| 2. Social Profitability (SP): H = E – (F + G); | | | |  | 76,357,729 | |
| 3. Private Cost Ratio : PCR = C/(A – B); | | | |  | 0.663 | |
| 4. Domestic Resource Cost Ratio : DRCR = G / (E – F); | | | | | 0.483 | |
| 5. Output Transfer : OT = A – E; | | | |  | -29,030,628 | |
| 6. Nominal Protection Coefficient on Tradable Output : NPCO = A/E; | | | | | 0.876 | |
| 7. Input Transfer : IT = B – F; | | |  |  | 5,192,273 | |
| 8. Nominal Protection Coefficient on Tradable Input : NPCI = B / F; | | | | | 1.060 | |
| 9. Transfer factor : FT = C – G; | | | |  | 3,910,736 | |
| 10. Effective Protection Coefficient : EPC = (A – B) / (E – F); | | | | | 0.768 | |
| 11. Net Transfer : NT = D – H; | | |  |  | -38,133,637 | |
| 12. Profitability Coefficient LPC = D/H; | | | |  | 0.501 | |
| 13. Subsidy Ratio to Producer : SRP = L/E. | | | |  | -0.163 | |

| S3c. Results of the Policy Analysis Matrix (PAM) analysis of potato farming in Wonosobo Regency, Central Java Province, Dry Season 2019 | | | | |
| --- | --- | --- | --- | --- |
| Variables | Revenue | Cost | | Profit |
|  |  | *tradable input* | *Domestic factor cost* |  |
| Private Price | 208,000,000 | 70,889,352 | 63,300,007 | 73,810,641 |
| Social Pricing | 246,376,000 | 60,376,952 | 59,353,005 | 126,646,043 |
| Divergence and policy impacts | -38,376,000 | 10,512,400 | 3,947,002 | -52,835,402 |
| 1. Private Profitability (PP): D = A – (B + C); | | |  | 73,810,641 |
| 2. Social Profitability (SP): H = E – (F + G); | | |  | 126,646,043 |
| 3. Private Cost Ratio : PCR = C/(A – B); | | |  | 0.462 |
| 4. Domestic Resource Cost Ratio : DRCR = G / (E – F); | | | | 0.319 |
| 5. Output Transfer : OT = A – E; | | |  | -38,376,000 |
| 6. Nominal Protection Coefficient on Tradable Output : NPCO = A/E; | | | | 0.844 |
| 7. Input Transfer : IT = B – F; | |  |  | 10,512,400 |
| 8. Nominal Protection Coefficient on Tradable Input : NPCI = B / F; | | | | 1.174 |
| 9. Transfer factor : FT = C – G; | | |  | 3,947,002 |
| 10. Effective Protection Coefficient : EPC = (A – B) / (E – F); | | | | 0.737 |
| 11. Net Transfer : NT = D – H; | |  |  | -52,835,402 |
| 12. Profitability Coefficient LPC = D/H; | | |  | 0.583 |
| 13. Subsidy Ratio to Producer : SRP = L/E. | | |  | -0.214 |

| S3d. Results of the Policy Analysis Matrix (PAM) analysis of potato farming in Wonosobo Regency, Central Java Province, Rain Season 2019-2020 | | | | |
| --- | --- | --- | --- | --- |
| Variables | Revenue | Cost | | Profit |
|  |  | *tradable input* | *Domestic factor cost* |  |
| Private price | 170,360,500 | 77,628,186 | 61,582,039 | 31,150,275 |
| Social Price | 212,973,100 | 63,882,451 | 56,968,298 | 92,122,351 |
| Policy and divergence impacts | -42,612,600 | 13,745,735 | 4,613,741 | -60,972,076 |
| 1. Private Profitability (PP): D = A – (B + C); | | |  | 31,150,275 |
| 2. Social Profitability (SP): H = E – (F + G); | | |  | 92,122,351 |
| 3. Private Cost Ratio : PCR = C/(A – B); | | |  | 0.664 |
| 4. Domestic Resource Cost Ratio : DRCR = G / (E – F); | | | | 0.382 |
| 5. Output Transfer : OT = A – E; | | |  | -42,612,600 |
| 6. Nominal Protection Coefficient on Tradable Output : NPCO = A/E; | | | | 0.800 |
| 7. Input Transfer : IT = B – F; | |  |  | 13,745,735 |
| 8. Nominal Protection Coefficient on Tradable Input : NPCI = B / F; | | | | 1,215 |
| 9. Transfer factor : FT = C – G; | | |  | 4,613,741 |
| 10. Effective Protection Coefficient : EPC = (A – B) / (E – F); | | | | 0.622 |
| 11. Net Transfer : NT = D – H; | |  |  | -60,972,076 |
| 12. Profitability Coefficient LPC = D/H; | | |  | 0.338 |
| 13. Subsidy Ratio to Producer : SRP = L/E. | | |  | -0.286 |

| S3e. Results of the Policy Analysis Matrix (PAM) analysis of potato farming in Pasuruan Regency, East Java Province, Dry Season 2019 | | | | |
| --- | --- | --- | --- | --- |
| Variables | Revenue | Cost | | Profit |
|  |  | *tradable input* | *Domestic factor cost* |  |
| Private price | 151,125,000 | 63,934,677 | 52,077,158 | 35,113,165 |
| Social Price | 182,734,500 | 60,523,377 | 50,755,637 | 71,455,486 |
| Divergence and policy impacts | -31,609,500 | 3,411,300 | 1,321,522 | -36,342,322 |
| 1. Private Profitability (PP): D = A – (B + C); | | |  | 35,113,165 |
| 2. Social Profitability (SP): H = E – (F + G); | | |  | 71,455,486 |
| 3. Private Cost Ratio : PCR = C/(A – B); | | |  | 0.597 |
| 4. Domestic Resource Cost Ratio : DRCR = G / (E – F); | | | | 0.415 |
| 5. Output Transfer : OT = A – E; | | |  | -31,609,500 |
| 6. Nominal Protection Coefficient on Tradable Output : NPCO = A/E; | | | | 0.827 |
| 7. Input Transfer : IT = B – F; | |  |  | 3,411,300 |
| 8. Nominal Protection Coefficient on Tradable Input : NPCI = B / F; | | | | 1.056 |
| 9. Transfer factor : FT = C – G; | | |  | 1,321,522 |
| 10. Effective Protection Coefficient : EPC = (A – B) / (E – F); | | | | 0.713 |
| 11. Net Transfer : NT = D – H; | |  |  | -36,342,322 |
| 12. Profitability Coefficient LPC = D/H; | | |  | 0.491 |
| 13. Subsidy Ratio to Producer : SRP = L/E. | | |  | -0.199 |

| S3f. The results of the Policy Analysis Matrix analysis of potato farming in Pasuruan Regency,  East Java Province, Rainy Season 2019-2020 | | | | |
| --- | --- | --- | --- | --- |
| Variables | Revenue | Cost | | Profit |
|  |  | *tradable input* | *Domestic factor cost* |  |
| Private price | 142,462,500 | 66,269,290 | 43,902,602 | 32,290,608 |
| Social Price | 184,140,150 | 59,439,850 | 42,568,627 | 82,131,673 |
| Divergence and policy impacts | -41,677,650 | 6,829,440 | 1,333,974 | -49,841,064 |
| 1. Private Profitability (PP): D = A – (B + C); | | |  | 32,290,608 |
| 2. Social Profitability (SP): H = E – (F + G); | | |  | 82,131,673 |
| 3. Private Cost Ratio : PCR = C/(A – B); | | |  | 0.576 |
| 4. Domestic Resource Cost Ratio : DRCR = G / (E – F); | | | | 0.341 |
| 5. Output Transfer : OT = A – E; | | |  | -41,677,650 |
| 6. Nominal Protection Coefficient on Tradable Output : NPCO = A/E; | | | | 0.774 |
| 7. Input Transfer : IT = B – F; | |  |  | 6,829,440 |
| 8. Nominal Protection Coefficient on Tradable Input : NPCI = B / F; | | | | 1.115 |
| 9. Transfer factor : FT = C – G; | | |  | 1,333,974 |
| 10. Effective Protection Coefficient : EPC = (A – B) / (E – F); | | | | 0.611 |
| 11. Net Transfer : NT = D – H; | |  |  | -49,841,064 |
| 12. Profitability Coefficient LPC = D/H; | | |  | 0.393 |
| 13. Subsidy Ratio to Producer : SRP = L/E. | | |  | -0.271 |

| S3g. The results of the Policy Analysis Matrix (PAM) analysis of potato businesses in Tanah Karo Regency, North Sumatra, Dry Season 2019 | | | | |
| --- | --- | --- | --- | --- |
| Variables | Revenue | Cost | | Profit |
|  |  | *tradable input* | *Domestic factor cost* |  |
| Private price | 130,147,500 | 66,076,451 | 45,146.062 | 18,924,987 |
| Social Price | 174,103,500 | 56,618,786 | 43,742,979 | 73,741,736 |
| Divergence and policy impacts | -43,956,000 | 9,457,665 | 1,403,084 | -54,816,749 |
| 1. Private Profitability (PP): D = A – (B + C); | | |  | 18,924,987 |
| 2. Social Profitability (SP): H = E – (F + G); | | |  | 73,741,736 |
| 3. Private Cost Ratio : PCR = C/(A – B); | | |  | 0.705 |
| 4. Domestic Resource Cost Ratio : DRCR = G / (E – F); | | | | 0.372 |
| 5. Output Transfer : OT = A – E; | | |  | -43,956,000 |
| 6. Nominal Protection Coefficient on Tradable Output : NPCO = A/E; | | | | 0.748 |
| 7. Input Transfer : IT = B – F; | |  |  | 9,457,665 |
| 8. Nominal Protection Coefficient on Tradable Input : NPCI = B / F; | | | | 1.167 |
| 9. Transfer factor : FT = C – G; | | |  | 1,403,084 |
| 10. Effective Protection Coefficient : EPC = (A – B) / (E – F); | | | | 0.545 |
| 11. Net Transfer : NT = D – H; | |  |  | -54,816,749 |
| 12. Profitability Coefficient LPC = D/H; | | |  | 0.257 |
| 13. Subsidy Ratio to Producer : SRP = L/E. | | |  | -0.315 |

| S3h. Results of the Policy Analysis Matrix (PAM) analysis of potato farming in Tanah Karo Regency, North Sumatra Province, Rain Season, 2019-2020 | | | | |
| --- | --- | --- | --- | --- |
| Variables | Revenue | Cost | | Profit |
|  |  | *tradable input* | *Domestic factor cost* |  |
| Private price | 151,125,000 | 67,717,288 | 45,756,867 | 37,650,846 |
| Social Price | 195,748,800 | 57,714,390 | 44,640,356 | 93,394,054 |
| Policy and divergence impacts | -44,623,800 | 10.002.898 | 1,116,511 | -55,743,208 |
| 1. Private Profitability (PP): D = A – (B + C); | | |  | 37,650,846 |
| 2. Social Profitability (SP): H = E – (F + G); | | |  | 93,394,054 |
| 3. Private Cost Ratio : PCR = C/(A – B); | | |  | 0.549 |
| 4. Domestic Resource Cost Ratio : DRCR = G / (E – F); | | | | 0.323 |
| 5. Output Transfer : OT = A – E; | | |  | -44,623,800 |
| 6. Nominal Protection Coefficient on Tradable Output : NPCO = A/E; | | | | 0.772 |
| 7. Input Transfer : IT = B – F; | |  |  | 10.002.898 |
| 8. Nominal Protection Coefficient on Tradable Input : NPCI = B / F; | | | | 1.173 |
| 9. Transfer factor : FT = C – G; | | |  | 1,116,511 |
| 10. Effective Protection Coefficient : EPC = (A – B) / (E – F); | | | | 0.604 |
| 11. Net Transfer : NT = D – H; | |  |  | -55,743,208 |
| 12. Profitability Coefficient LPC = D/H; | | |  | 0.403 |
| 13. Subsidy Ratio to Producer : SRP = L/E. | | |  | -0.285 |

| S3i. Results of the Policy Analysis Matrix (PAM) analysis of potato farming in Solok Regency, West Sumatra Province, Dry Season, 2019 | | | | |
| --- | --- | --- | --- | --- |
| Variables | Revenue | Cost | | Profit |
|  |  | *tradable input* | *Domestic factor cost* |  |
| Private price | 168,618,000 | 66,774,209 | 61,670,134 | 40,173,656 |
| Social Price | 203,329,680 | 56,472,034 | 56,486,434 | 90,371,212 |
| Policy and divergence impacts | -34,711,680 | 10,302,175 | 5,183,700 | -50,197,555 |
| 1. Private Profitability (PP): D = A – (B + C); | | |  | 40,173,656 |
| 2. Social Profitability (SP): H = E – (F + G); | | |  | 90,371,212 |
| 3. Private Cost Ratio : PCR = C/(A – B); | | |  | 0.606 |
| 4. Domestic Resource Cost Ratio : DRCR = G / (E – F); | | | | 0.385 |
| 5. Output Transfer : OT = A – E; | | |  | -34,711,680 |
| 6. Nominal Protection Coefficient on Tradable Output : NPCO = A/E; | | | | 0.829 |
| 7. Input Transfer : IT = B – F; | |  |  | 10,302,175 |
| 8. Nominal Protection Coefficient on Tradable Input : NPCI = B / F; | | | | 1.182 |
| 9. Transfer factor : FT = C – G; | | |  | 5,183,700 |
| 10. Effective Protection Coefficient : EPC = (A – B) / (E – F); | | | | 0.693 |
| 11. Net Transfer : NT = D – H; | |  |  | -50,197,555 |
| 12. Profitability Coefficient LPC = D/H; | | |  | 0.445 |
| 13. Subsidy Ratio to Producer : SRP = L/E. | | |  | -0.247 |

| S3j. The results of the Policy Analysis Matrix (PAM) analysis of potato farming in Solok Regency, West Sumatra Province, Rain Season 2019-2020 | | | | | | | | | |
| --- | --- | --- | --- | --- | --- | --- | --- | --- | --- |
| Variables | | Revenue | Cost | | | | Profit | | |
|  |  |  | *tradable input* | | *Domestic factor cost* | |  |  |  |
| Private price | | 153,872,000 | 64,412,033 | | 60,420,465 | | 29,039,502 | | |
| Social Price | | 178,718,080 | 56,472,034 | | 56,486,434 | | 65,759,612 | | |
| Policy and divergence impacts | | -24,846,080 | 7,939,999 | | 3,934,031 | | -36,720,110 | | |
| 1. Private Profitability (PP): D = A – (B + C); | | | | |  | | 29,039,502 | | |
| 2. Social Profitability (SP): H = E – (F + G); | | | | |  | | 65,759,612 | | |
| 3. Private Cost Ratio : PCR = C/(A – B); | | | | |  | | 0.675 | | |
| 4. Domestic Resource Cost Ratio : DRCR = G / (E – F); | | | | | | | 0.462 | | |
| 5. Output Transfer : OT = A – E; | | | | |  | | -24,846,080 | | |
| 6. Nominal Protection Coefficient on Tradable Output : NPCO = A/E; | | | | | | | 0.861 | | |
| 7. Input Transfer : IT = B – F; | | |  | |  | | 7,939,999 | | |
| 8. Nominal Protection Coefficient on Tradable Input : NPCI = B / F; | | | | | | | 1.141 | | |
| 9. Transfer factor : FT = C – G; | | | | |  | | 3,934,031 | | |
| 10. Effective Protection Coefficient : EPC = (A – B) / (E – F); | | | | | | | 0.732 | | |
| 11. Net Transfer : NT = D – H; | | |  | |  | | -36,720,110 | | |
| 12. Profitability Coefficient LPC = D/H; | | | | |  | | 0.442 | | |
| 13. Subsidy Ratio to Producer : SRP = L/E. | | | | |  | | -0.205 | | |
| S3k. Results of the Policy Analysis Matrix (PAM) analysis of potato farming in Kerinci Regency, Jambi Province, Dry Season, 2019 | | | | | | | | |  |
| Variables | Revenue | | | Cost | | | | Profit |  |
|  |  |  |  | *tradable input* | | *Domestic factor cost* | |  |  |
| Private price | 170,737,500 | | | 80,074,073 | | 49,130,579 | | 41,532,849 |  |
| Social Price | 207,625,500 | | | 67,850,873 | | 47,473,447 | | 92,301,180 |  |
| Policy and divergence impacts | -36,888,000 | | | 12,223,200 | | 1,657,132 | | -50,768,332 |  |
| 1. Private Profitability (PP): D = A – (B + C); | | | | | |  | | 41,532,849 |  |
| 2. Social Profitability (SP): H = E – (F + G); | | | | | |  | | 92,301,180 |  |
| 3. Private Cost Ratio : PCR = C/(A – B); | | | | | |  | | 0.542 |  |
| 4. Domestic Resource Cost Ratio : DRCR = G / (E – F); | | | | | | | | 0.340 |  |
| 5. Output Transfer : OT = A – E; | | | | | |  | | -36,888,000 |  |
| 6. Nominal Protection Coefficient on Tradable Output : NPCO = A/E; | | | | | | | | 0.822 |  |
| 7. Input Transfer : IT = B – F; | | | |  | |  | | 12,223,200 |  |
| 8. Nominal Protection Coefficient on Tradable Input : NPCI = B / F; | | | | | | | | 1.180 |  |
| 9. Transfer factor : FT = C – G; | | | | | |  | | 1,657,132 |  |
| 10. Effective Protection Coefficient : EPC = (A – B) / (E – F); | | | | | | | | 0.649 |  |
| 11. Net Transfer : NT = D – H; | | | |  | |  | | -50,768,332 |  |
| 12. Profitability Coefficient LPC = D/H; | | | | | |  | | 0.450 |  |
| 13. Subsidy Ratio to Producer : SRP = L/E. | | | | | |  | | -0.245 |  |

| S3l. Results of the Policy Analysis Matrix (PAM) analysis of potato farming in Kerinci Regency Jambi Province, Rain Season, 2019-2020 | | | | |
| --- | --- | --- | --- | --- |
| Variables | Revenue | Cost | | Profit |
|  |  | *Tradeable input costs* | *Domestic factor cost* |  |
| Private price | 152,812,500 | 82,520,286 | 46,694,696 | 23,597,518 |
| Social Price | 178,987,500 | 68,116,203 | 44,482,918 | 66,388,379 |
| Policy and divergence impacts | -26,175,000 | 14,404,084 | 2,211,778 | -42,790,862 |
| 1. Private Profitability (PP): D = A – (B + C); | | |  | 23,597,518 |
| 2. Social Profitability (SP): H = E – (F + G); | | |  | 66,388,379 |
| 3. Private Cost Ratio : PCR = C/(A – B); | | |  | 0.664 |
| 4. Domestic Resource Cost Ratio : DRCR = G / (E – F); | | | | 0.401 |
| 5. Output Transfer : OT = A – E; | | |  | -26,175,000 |
| 6. Nominal Protection Coefficient on Tradable Output : NPCO = A/E; | | | | 0.854 |
| 7. Input Transfer : IT = B – F; | |  |  | 14,404,084 |
| 8. Nominal Protection Coefficient on Tradable Input : NPCI = B / F; | | | | 1,211 |
| 9. Transfer factor : FT = C – G; | | |  | 2,211,778 |
| 10. Effective Protection Coefficient : EPC = (A – B) / (E – F); | | | | 0.634 |
| 11. Net Transfer : NT = D – H; | |  |  | -42,790,862 |
| 12. Profitability Coefficient LPC = D/H; | | |  | 0.355 |
| 13. Subsidy Ratio to Producer : SRP = L/E. | | |  | -0.239 |
